# Synergistic Effect of La and TiB_2_ Particles on Grain Refinement in Aluminum Alloy

**DOI:** 10.3390/ma15020600

**Published:** 2022-01-13

**Authors:** Lili Zhang, Yan Song, Linjie Yang, Jiuzhou Zhao, Jie He, Hongxiang Jiang

**Affiliations:** 1 Shi-Changxu Innovation Centre for Advanced Materials, Institute of Metal Research, Chinese Academy of Sciences, Shenyang 110016, China; ysong16b@imr.ac.cn (Y.S.); ljyang19s@imr.ac.cn (L.Y.); jhe@imr.ac.cn (J.H.); hxjiang@imr.ac.cn (H.J.); 2 School of Materials Science and Engineering, University of Science and Technology of China, Shenyang 110016, China

**Keywords:** grain refinement, microstructure, segregation, heterogeneous nucleation, metals and alloys

## Abstract

Synergistic effect of TiB_2_ (in form of Al-5Ti-1B) and La on grain refining results in Al-2Cu alloy was investigated. α-Al grains are significantly refined by Al-5Ti-1B. When trace La is added to the melt, further refinement is exhibited. Average grain size and nucleation undercooling of α-Al reduce first and then almost remain unchanged with La addition. Satisfactory grain refining result achieves when La addition level reaches 600 ppm. When more than 600 ppm La is added to the melt, La-rich particles form and the effect of solute La left in matrix on the microstructure almost no longer changes. Theoretical calculation results demonstrate that solute La segregates to Al melt/TiB_2_ particles interface along with Ti and Cu prior to α-Al nucleation and the synergistic effect of La and TiB_2_ particles on grain refinement mainly attributes to the enhancement in the potency of TiB_2_ particles to heterogeneously nucleate α-Al by trace La addition.

## 1. Introduction

Grain refinement contributes to the enhancement in mechanical properties of alloys. Inoculation by adding master alloys (such as Al-Zr, Al-Cr, Al-B, and Al-Ti-B) to melt has been widely employed to improve the microstructures of aluminum alloys [1,2,3]. Among them, Al-5Ti-1B (mass percent, the same as below unless otherwise specified) master alloy is the most widely used grain refiner. Since the introduction of Al-Ti-B, much work has been done to explore microstructure formation in aluminum alloys under the effect of grain refiners [4,5,6,7,8,9,10,11,12,13]. It is now acknowledged that size distribution of TiB_2_ and solute Ti concentration play an important role in grain refinement.

TiB_2_ particles heterogeneously nucleate α-Al grains, while solute Ti affects the potency of TiB_2_ to heterogeneously nucleate α-Al and restricts growth of α-Al grains, which is quantitatively described as growth restriction factor $Q_{\mathrm{Ti}}\left(=m_{\mathrm{Ti}}c_{\mathrm{Ti}}^{0}\left(k_{\mathrm{Ti}}-1\right)\right)$, $c_{\mathrm{Ti}}^{0}$, $k_{\mathrm{Ti}}$ = 7.8, and $m_{\mathrm{Ti}}$ = 33.3 K/wt% are respectively the solute Ti concentration, equilibrium partition coefficient, and liquidus slope in Al-Ti phase diagram [9]. When solute Ti concentration is low, contact angle between α-Al and TiB_2_ particles is large and restriction effect of solute Ti on α-Al growth is small. This results in a weak grain refinement result and only columnar grain structure is refined. Grain refinement result enhances and columnar-to-equiaxed transition is present when solute Ti concentration is high.

In spite of great progress in the investigation of microstructure formation in inoculated aluminum alloys, limited grain refinement potency of Al-5Ti-1B cannot meet the requirement for manufacturing high-quality aluminum alloys. Enhancement in grain refinement of Al-5Ti-1B is quite necessary in industry. La, one of the most economical rare elements, has attracted much attention since the 1990s due to its significant effect on grain refinement of matrix [14,15] and modification of phases in aluminum alloys [14,16,17,18]. Recently, attention is shifting to the synergistic effect of La and other elements, such as B [19], Mg [20], and Sr [21], on microstructures of aluminum alloys. Our latest work demonstrates that only a few hundred ppm of La is enough to improve the microstructures [22,23,24]. Considering the wide use of Al-5Ti-1B and great potentials of La in improving microstructure, it is necessary to explore the combined effect of TiB_2_ particles and trace La on microstructure evolution of aluminum alloys.

## 2. Materials and Methods

High-purity Al (99.99%), Cu (99.999%), and La (99.99%) and Al-5Ti-1B prepared in our lab were used as raw materials. Al-2Cu and Al-10La alloys are respectively prepared as follows: first, melting high-purity Al and heating to 1003 K; then, adding high-purity Cu or La to melt and holding the melt at 1003 K for 30 min; finally, solidifying the melt to obtain Al-2Cu and Al-10La alloys. The process for grain refinement was as follows: first, melting Al-2Cu alloy and heating to 1003 K; then, adding trace La to melt in form of Al-10La and holding the melt at 1003 K for 20 min; after that, adding Al-5Ti-1B to melt and holding the melt at 1003 K for 10 min; finally, solidifying the melt to form an ingot with a diameter of 20 mm and height of 40 mm. Temperature at the center of cross-section approximately 15 mm from bottom was measured using tungsten–rhenium thermocouple of 0.2 mm in radius. The cooling rate of the melt is about 20 K/s.

Process described in reference [23] was employed to prepare metallographic specimens. Microstructures of α-Al were examined by using Zeiss optical microscope (Carl Zeiss AG, Germany) with polarized light after the specimens were ground, polished electrolytically for about 20 s at 50 V in a reagent (5 vol% HClO_4_ in ethanol solution) and anodized for about 120 s at 20 V in Barker’s reagent (2.5 vol% HBF_4_ in distilled water). Sizes of α-Al grains, obtained from the same center region of the cross-section, were determined by quantitative metallographic analysis using SISC IAS V8.0 software. Scanning electron microscopy (SEM, Quanta 450, FEI, Hillsboro, FL, USA) equipped with an energy dispersive X-ray spectroscopy (EDS) was also used to characterize the microstructure. Differential thermal analyzer (DTA, Beijing Jingyi gaoke Instrument Co., Ltd., Beijing, China) experiments were performed with heating/cooling rate of 10 K/min. Transmission electron microscope (TEM) is also employed to characterize microstructures. Specimens for TEM investigation were first cut into discs with a diameter of 3 mm and thickness of 0.5 mm and ground to a thickness of about 50 μm. Discs were then dimpled and ion-beam-thinned by using Gatan Precision Ion Polishing System (Gatan 691, Gatan, Pleasanton, CA, USA) under the conditions of 1~5 kV and an incident angle of 3~8°. TEM analyses were performed by using Tecnai G2 20 (FEI, Hillsboro, FL, USA).

## 3. Results

**Figure 1:**
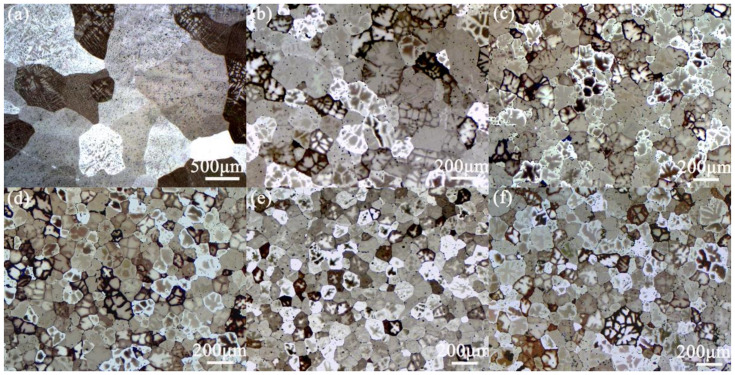
Microstructures of Al-2Cu alloy (**a**) without addition of inoculant and by adding 0.4% Al-5Ti-1B + La addition of (**b**) 0%, (**c**) 0.02%, (**d**) 0.06%, (**e**) 0.08%, and (**f**) 0.10%.

**Figure 2:**
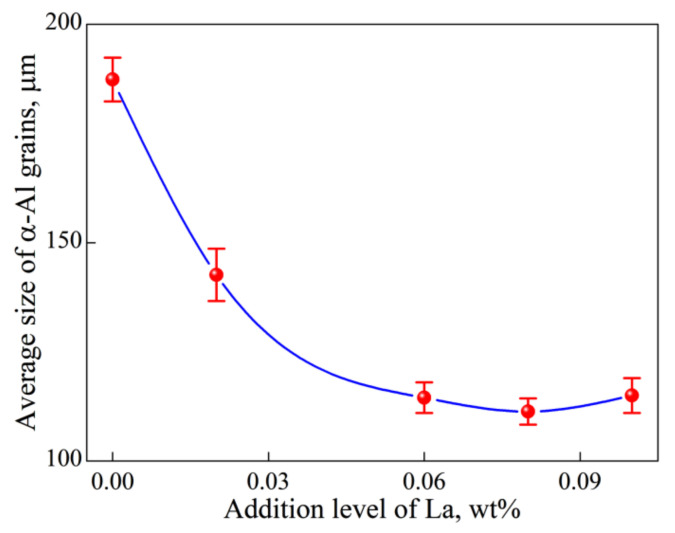
Average size of α-Al grains in Al-2Cu alloy by adding 0.4% Al-5Ti-1B vs. La addition level.

Figure 1 and Figure 2 respectively show microstructures and average grain sizes of Al-2Cu alloy without addition of inoculant and by adding 0.4% Al-5Ti-1B + trace La. α-Al grains are significantly refined by 0.4% Al-5Ti-1B with the average grain size of α-Al decreasing to 187 ± 5 μm from 654 ± 4 μm. Microstructure exhibits a further refinement when La is introduced and grain size decreases first and then almost keeps unchanged with La addition.

**Figure 3:**
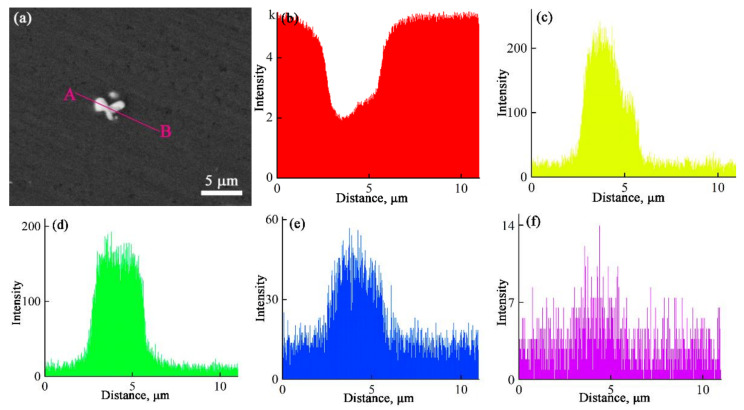
SEM image and EDS line-scanning results for Al-2Cu alloy inoculated with 0.4% Al-5Ti-1B + 0.08% La. (**a**) back-scattered electron image; (**b**–**f**) distributions of Al, Cu, La, Ti, and B along line A and B.

Figure 3 shows the SEM image and EDS line-scanning results for Al-2Cu alloy inoculated with 0.4% Al-5Ti-1B + 0.08% La. The results demonstrate that trace La enriches at the surface of TiB_2_ particles along with solutes Cu and Ti.

**Figure 4:**
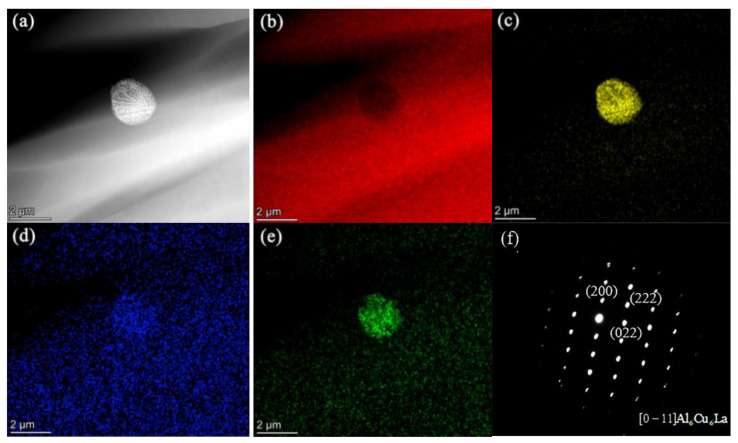
(**a**) TEM image, elemental maps of (**b**) Al, (**c**) Cu, (**d**) Ti, and (**e**) La and (f) selected area electron diffraction pattern in the zone axis of [0–11]Al_6_Cu_6_La of the particles consisted of Al, Cu, and La in Al-2Cu alloy by adding 0.4% Al-5Ti-1B + 0.08% La.

TEM elemental map results demonstrate that La-rich particles (Al_6_Cu_6_La) form when La addition reaches 0.08%, as shown in Figure 4.

**Figure 5:**
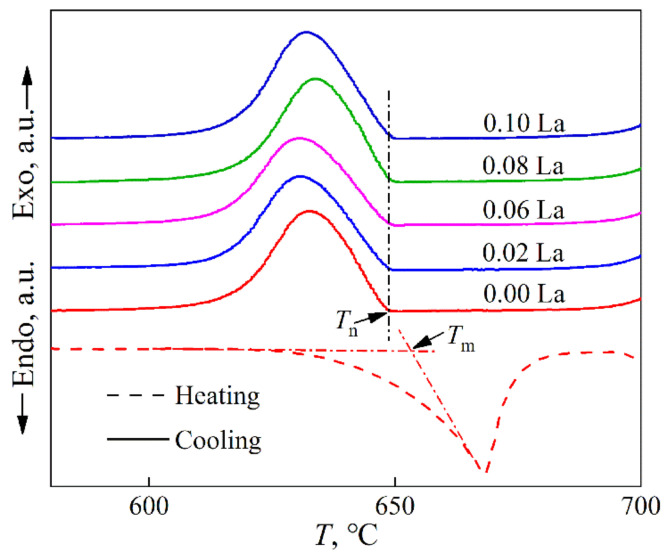
DTA heating and cooling curves for Al-2Cu alloy inoculated with 0.4% Al-5Ti-1B vs. La addition level. *T*_n_ and *T*_m_ are respectively the nucleation temperature and melting temperature. Heating/cooling rate is 10 K/min.

Figure 5 shows DTA curves of Al-2Cu alloy by adding 0.4% Al-5Ti-1B + trace La. Onset temperatures of exothermic and endothermic peaks are respectively the nucleation temperature *T*_n_ and melting temperature *T*_m_ of α-Al. The nucleation undercooling of α-Al Δ*T*_n_ ( = *T*_m_ − *T*_n_) can be determined by DTA curves. Δ*T*_n_ for Al-2Cu alloy by adding 0.4% Al-5Ti-1B is 4.4 °C. It decreases further with La concentration less than 0.06% and then almost changes little with La addition. Figure 2, Figure 3, Figure 4 and Figure 5 demonstrate that La and TiB_2_ mainly affect nucleation process of α-Al, and the effect increases with La addition less than 0.06%. When more than 0.06% La is added, La-rich particles form and the effect of solute La left in matrix on the microstructure almost no longer changes.

## 4. Discussion

As demonstrated in Figure 3, solutes La, Ti, and Cu segregate to Al(L)/TiB_2_(S) interface. In the following discussion, we will first investigate the reason for solute segregation and then analyze its effect on grain refinement result of Al-5Ti-1B.

### 4.1. Segregation of La to Al Melt/TiB_2_ Particles Interface along with Ti and Cu

For Al-2Cu alloy melt by adding La and Al-5Ti-1B, whether element i (i represents La, Ti, or Cu) enriches the melt/TiB_2_ particles interface or not depends on the relationship between interfacial energies of pure solute i(L)/TiB_2_(S) $\sigma_{i\left(L\right){\mathrm{TiB}}_{2}\left(s\right)}^{0}$ and Al(L)/TiB_2_(S) $\sigma_{\mathrm{Al}\left(L\right){\mathrm{TiB}}_{2}\left(s\right)}^{0}=0.853\;J/m^{2}$ and between the entropies of fusion of solute i $\Delta S_{m}^{i}$ and Al $\Delta S_{m}^{\mathrm{Al}}$, and the interaction energy parameters of solute i atom and Al atom in Al-i solution Ω_Al-i_.

$\sigma_{i\left(L\right){\mathrm{TiB}}_{2}\left(s\right)}^{0}$ can be calculated by Equation (1) [25]:

(1) $$\sigma_{{}_{{i(L)/\mathrm{TiB}}_{2}\left(S\right)}}^{0}=\frac{\frac{0.364\left(2\Omega_{i-B}^{}+\Omega_{i-\mathrm{Ti}}^{}-\Delta_{f}H_{{\mathrm{TiB}}_{2}}\right)}{3}+\frac{0.310f\cdot f_{b}^{1/3}\left(\Delta_{m}H_{\mathrm{Ti}}+2\Delta_{m}H_{B}\right)}{3}+(3.5\pm 1)T}{\omega_{{i(L)/\mathrm{TiB}}_{2}\left(S\right)}}$$

where $\Delta_{f}H_{{\mathrm{TiB}}_{2}}=323800\;J/\mathrm{mol}$ is the heat of formation for TiB_2_, $\Delta_{m}H_{\mathrm{Ti}}=14146\;J/\mathrm{mol}$ and $\Delta_{m}H_{B}=50200\;J/\mathrm{mol}$ are respectively the enthalpies of fusion of Ti and B, *f*_b_ = 0.74 is the bulk packing factor, *f* = 1.09 [25], Ω_i-B_ and Ω_i-Ti_ are respectively the interaction energy parameters of i-B melt and i-Ti melt. $\omega_{i\left(L\right)/{\mathrm{TiB}}_{2}\left(s\right)}\approx \sqrt{\omega_{i\left(L\right)}\omega_{\mathrm{Ti}\left(L\right)}}$ is the molar area of i(L)/TiB_2_(S) interface, $\omega_{i\left(L\right)}=f{\left(N_{a}\right)}^{1/3}V_{i\left(L\right)}^{2/3}$ is the molar area of i melt, $N_{a}=6.02\times 10^{23}\;{\mathrm{mol}}^{-1}$ is the Avogadro’s number, $V_{\mathrm{La}\left(L\right)}=2.33\times 10^{-5}\;m^{3}/\mathrm{mol}$, $V_{\mathrm{Ti}\left(L\right)}=1.16\times 10^{-5}\;m^{3}/\mathrm{mol}$, and $V_{\mathrm{Cu}\left(L\right)}=7.94\times 10^{-6}\;m^{3}/\mathrm{mol}$ are the molar volumes of La, Ti, and Cu melt, respectively [26].

Interaction energy parameter Ω_j-i_ (j represents Ti, B or Al, j ≠ i) is determined by Equation (2) [25]:

(2) $$\Omega_{j-i}{\left(1-x_{i}\right)}^{2}=R_{g}T\ln\gamma_{i}$$

where $\gamma_{i}$ is the activity coefficient of i, which is obtained by using Wilson equation $\ln\gamma_{i}=1-\ln\left(1-x_{j}A_{j/i}\right)-x_{i}/\left(1-x_{j}A_{j/i}\right)-x_{j}\left(1-A_{i/j}\right)/\left(1-x_{i}A_{i/j}\right)$. . *A*_i/j_ and *A*_j/i_ are the Wilson parameters [27].

**Figure 6:**
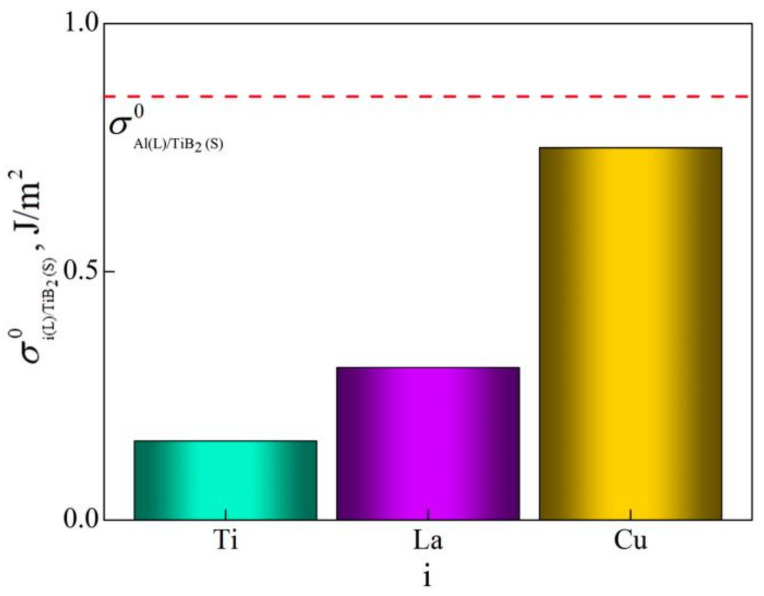
Interfacial energy $\sigma_{{}_{{i(L)/\mathrm{TiB}}_{2}\left(S\right)}}^{0}$ of i(L)/TiB_2_(S) at 660 °C.

Calculated results for $\sigma_{i\left(L\right){\mathrm{TiB}}_{2}\left(s\right)}^{0}$ at 660 °C are shown in Figure 6. It is demonstrated that the values of $\sigma_{i\left(L\right){\mathrm{TiB}}_{2}\left(s\right)}^{0}$ are less than $\sigma_{\mathrm{Al}\left(L\right){\mathrm{TiB}}_{2}\left(s\right)}^{0}$, indicating that solutes La and Cu tend to enrich Al(L)/TiB_2_(S) interface along with solute Ti.

The mole fraction $x_{i}^{\mathrm{In}}$ of element i at Al(L)/TiB_2_(S) interface depends on $x_{i}^{0}$ in the melt according to the following Equation (3) [25]:

(3) $$\begin{array}{c} \ln\left[\left(\frac{x_{i}^{\mathrm{In}}}{1-x_{i}^{\mathrm{In}}}\right)/\left(\frac{x_{i}^{0}}{1-x_{i}^{0}}\right)\right]=\frac{\frac{2\Omega_{\mathrm{Al}-i}}{Z}\left[Z_{L}\left(x_{i}^{\mathrm{In}}-x_{i}^{0}\right)-Z_{1}\left(x_{i}^{0}-0.5\right)\right]+\left(\Delta S_{m}^{\mathrm{Al}}-\Delta S_{m}^{i}\right)T}{R_{g}T}-\\ \frac{\omega_{{i(L)/\mathrm{TiB}}_{2}\left(S\right)}\left(\gamma_{{i(L)/\mathrm{TiB}}_{2}\left(S\right)}^{0}-\gamma_{{\mathrm{Al}(L)/\mathrm{TiB}}_{2}\left(S\right)}^{0}\right)}{R_{g}T} \end{array}$$

where *Z* = 12 and *Z*_L_ = 6 are respectively the atomic coordination numbers of the melt and interfacial monolayer; *Z*_1_ = 3 is the atomic coordination number to one of the adjacent layers, Ω_Al-Ti_ = −120,000 J/mol, $\Delta S_{m}^{i}$ and $\Delta S_{m}^{\mathrm{Al}}=11.47\;J/\left(\mathrm{mol}\cdot K\right)$ are respectively the entropies of fusion of i and Al ($\Delta S_{m}^{\mathrm{La}}=5.19\;J/\left(\mathrm{mol}\cdot K\right)$, $\Delta S_{m}^{\mathrm{Ti}}=7.288\;J/\left(\mathrm{mol}\cdot K\right)$ and $\Delta S_{m}^{\mathrm{Cu}}=9.768\;J/\left(\mathrm{mol}\cdot K\right)$ [25,28]).

**Figure 7:**
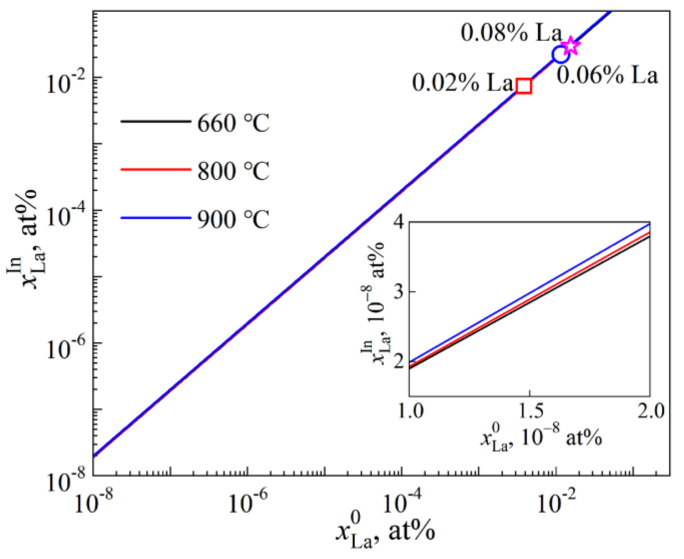
$x_{\mathrm{La}}^{\mathrm{In}}$ as a function of $x_{\mathrm{La}}^{0}$. Symbols present the points corresponding to the La addition level to melt.

Figure 7 shows the dependence of $x_{\mathrm{La}}^{\mathrm{In}}$ on $x_{\mathrm{La}}^{0}$. It is demonstrated that $x_{\mathrm{La}}^{\mathrm{In}}$ increases with $x_{\mathrm{La}}^{0}$ and the variation of $x_{\mathrm{La}}^{\mathrm{In}}$ with $x_{\mathrm{La}}^{0}$ is almost unaffected by temperature especially for a low $x_{\mathrm{La}}^{0}$. Temperature effect on i segregation is thus reasonable to neglect.

### 4.2. Effect of La and TiB_2_ on Grain Refinement of Al-2Cu Alloy

Considering that nucleation and growth processes of α-Al determine microstructure evolution of Al-2Cu alloy. The synergetic effects of La and TiB_2_ on grain refinement will be discussed from the above two aspects.

Restriction effect of i (i represents La or Cu) on the growth of α-Al grains can be calculated by Equation (4) [29]:

(4) $$Q_{i}=\left\{\begin{array}{l} m_{i}c_{i}^{0}\left(k_{i}-1\right),\;0<c_{i}^{0}\leq {c^{\prime }}_{\mathrm{iS}}\\ \frac{m_{i}c_{i}^{0}\left(k_{i}-1\right)\left({c^{\prime }}_{\mathrm{iL}}-c_{i}^{0}\right)k_{i}}{\left({c^{\prime }}_{\mathrm{iL}}-{c^{\prime }}_{\mathrm{iS}}\right)-\left(1-k_{i}\right)\left({c^{\prime }}_{\mathrm{iL}}-c_{i}^{0}\right)},\;{c^{\prime }}_{\mathrm{iS}}<c_{i}^{0}\leq {c^{\prime }}_{\mathrm{iL}} \end{array}\right.$$

where *k*_i_ and *m*_i_ are respectively the equilibrium partition coefficient and liquidus slope, $c_{\mathrm{iS}}^{\prime }$ is the i solubility of primary α-Al and $c_{\mathrm{iL}}^{\prime }$ is the eutectic composition.

According to Equation (4), $Q_{\mathrm{La}}^{\max}{|}_{0.05\%\mathrm{La}}=0.09\;K$ with *m*_La_ = −1.71 K/wt%, and *k*_La_ = 0.004 [30] is much less that *Q*_Ti_ = 2.54 K and *Q*_Cu_ = 5.64 K with *m*_Cu_ = −3.4 K/wt% and *k*_Cu_ = 0.17 [29], indicating that trace La effect on growth of α-Al grain is negligible. Thus, the addition of trace La and TiB_2_ particles mainly affects the nucleation process of α-Al.

When α-Al nucleates homogeneously, nucleation rate *I*_Hom_ of α-Al is described (Equation (5)):

(5) $$I_{\mathrm{Hom}}=10^{40}\exp\left(-\frac{16\pi \sigma_{\mathrm{Al}(L)/\alpha -\mathrm{Al}(S)}^{3}}{3k_{b}T_{\mathrm{nHom}}\Delta G_{\mathrm{VHom}}^{2}}\right)$$

where $\sigma_{\mathrm{Al}\left(L\right)/\alpha -\mathrm{Al}\left(S\right)}=0.158\;J/m^{2}$ is the interfacial energy of Al(L)/α-Al(S), *k*_b_ = 1.38 × 10^−23^ J/K is the Boltzmann’s constant, *T*_nHom_ is the homogeneous nucleation temperature, Δ*G*_Vhom_ = Δ*S*_V_Δ*T*_nHom_ is the driving force for homogeneous nucleation of α-Al with Δ*T*_nHom_, and Δ*S*_V_ = 1.11 × 10^6^ J/(m^3^·K) [8] respectively being the homogeneous nucleation undercooling and entropy of fusion per unit volume.

When 0.4% Al-5Ti-1B and trace La are added to the melt, α-Al nucleates on the substrates, just like Ni-base superalloys [31]. Heterogeneous nucleation rate *I*_Heter_ of α-Al is as follows (Equation (6)):

(6) $$I_{\mathrm{Heter}}=10^{40}\exp\left(-\frac{16\pi \sigma_{\mathrm{Al}(L)/\alpha -\mathrm{Al}(S)}^{3}}{3k_{b}T_{\mathrm{nHeter}}\Delta G_{\mathrm{VHeter}}^{2}}f\left(\theta \right)\right)$$

where *T*_nHeter_ is the heterogeneous nucleation temperature, Δ*G*_VHeter_ = Δ*S*_V_Δ*T*_nHeter_ is the driving force for heterogeneous nucleation of α-Al with Δ*T*_nHeter_ being the heterogeneous nucleation undercooling, $f\left(\theta \right)=\left(\cos^{3}\theta -3\cos\theta +2\right)/4$ is the catalytic factor, *θ* the contact angle between α-Al and substrates.

When nucleation of α-Al just starts, it is can be considered that *I*_Hom_ = *I*_Heter_ = 10^6^ m^−3^ s^−1^. *f*(*θ*) can be thus described as (Equation (7)):

(7) $$f\left(\theta \right)=\frac{T_{\text{nHeter}}\Delta T_{\text{nHeter}}^{2}}{T_{\text{nHom}}\Delta T_{\text{nHom}}^{2}}$$

By using the relation *T*_nHom_ = 1/3*T*_m_ [32], Equation (7) can be written as (Equation (8)):

(8) $$f\left(\theta \right)=\frac{27\left(T_{m}-\Delta T_{\mathrm{nHeter}}\right)\Delta T_{\mathrm{nHeter}}^{2}}{4T_{m}^{3}}$$

**Figure 8:**
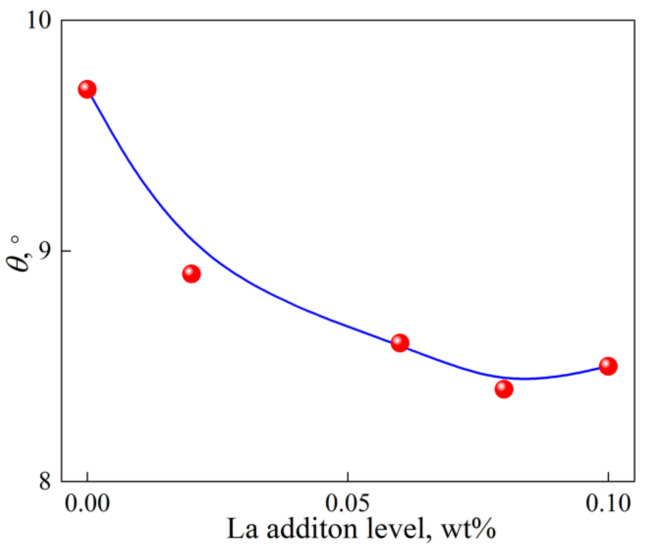
Calculated contact angle *θ* varied with trace La addition level.

The contact angle *θ* can thus be determined according to the experimental conditions by using Equation (8), as shown in Figure 8. When trace La is added to melt, *θ* decreases and heterogeneous nucleation of α-Al occurs at a smaller undercooling. *θ* shows a tendency of first decrease with La addition below 0.06% and then almost being unchanged with La addition. Combined with Section 4.1, it can be concluded that heterogeneous nucleation potency of TiB_2_ particles is enhanced by trace La addition due to solute segregation at Al(L)/TiB_2_ interface. Grain refinement result of Al-5Ti-1B is thus improved by trace La addition.

## 5. Conclusions

(1) Combinedly adding Al-5Ti-1B and trace La causes a further grain refinement result compared to the individual addition of Al-5Ti-1B. Average size of α-Al grains decreases first and then almost keeps unchanged with La addition. Satisfactory grain refinement result achieves when La addition reaches 600 ppm.
(2) Theoretical calculations were carried out to investigate the segregation of solute La to melt/TiB_2_ interface and segregation effect on grain refinement result of Al-5Ti-1B.
(3) Synergistic effect of La and TiB_2_ on grain refinement is mainly attributed to the enhancement in the heterogeneous nucleation potency of TiB_2_ particles for α-Al by La segregation to Al melt/TiB_2_ particles interface.

## Data Availability

Data is contained within the article.
